# Hazardous alcohol consumption in slow- and fast-privatized Russian industrial towns

**DOI:** 10.1038/s41598-024-62077-0

**Published:** 2024-05-22

**Authors:** Alexi Gugushvili, Aytalina Azarova, Darja Irdam, Lawrence King

**Affiliations:** 1 Department of Sociology and Human Geography, University of Oslo, Postboks 1096, Blindern, 0317 Oslo, Norway; 2 Department of Public Health and Primary Care, University of Cambridge, Worts Causeway, Cambridge, CB1 8RN UK; 3 Hall & Partners, Bankside 2, 90-100 Southwark Street, London, SE1 0SW UK; 4 Department of Economics, University of Massachusetts, Crotty Hall, 412 North Pleasant Street, Amherst, MA 01002 USA

**Keywords:** Hazardous drinking, Non-beverage alcohol, Russia, Transition, Demographic cohort study, Multilevel analysis, Risk factors, Predictive markers

## Abstract

Hazardous drinking, defined as the consumption of homemade, unofficially made alcohol and non-beverages, is prevalent and accounts for a high proportion of alcohol-related deaths in Russia. Individual-level characteristics are important explanations of hazardous drinking, but they are unlikely to explain spatial variation in this type of alcohol consumption. Areas that attracted insufficient attention in the research of hazardous drinking are the legacy of industrialization and the speed of economic reforms, mainly through the privatization policy of major enterprises in the 1990s. Applying mixed-effects logistic regressions to a unique dataset from 30 industrial towns in the European part of Russia, we find that in addition to individual-level characteristics such as gender, age, marital status, education, social isolation, labor market status, and material deprivation, the types of towns where informants’ relatives resided such as industrial structure and speed of privatization also accounted for the variance in hazardous alcohol consumption among both male and female populations of the analyzed towns.

## Introduction

Alcohol has been identified as the key mediator between the post-Soviet socio-economic transition and the unprecedented mortality crisis that Russia faced since the beginning of the 1990s^1–3^. Hazardous alcohol consumption in particular, both in terms of frequency of heavy drinking and in terms of the type of alcohol consumed, played a central role in causing detrimental morbidity and mortality consequences^4–6^, accounting, as some studies show, for more than 40 percent of deaths in the male population^7,8^.

So-called *samogon*, homemade alcohol, and ethanol-containing liquids have long been demonstrated as one of the riskiest types of consumed alcohol^9–11^. The high concentration of methanol or other impurities such as amyl alcohol or other fusel oils can be lethal even at very low doses, mostly due to the severe damage to the kidneys and toxic hepatitis that they cause^12,13^. The study of the composition of non-beverage alcohol consumed in Russia in 2015–2017 identified, in some of its types, a wide variety of impurities and admixtures that increased their toxicity^14^. *Samogon* is often fortified or deodorized with substances such as tobacco, sulphuric acid, gasoline, bird droppings, kerosene, burnt rubber, and even with diphenhydramine to increase the potency of alcohol, despite the obvious health risks^15,16^. Estimates suggest that in the 1990s and 2000s, the share of *samogon* and other unofficial types of alcohol constituted 30 to 50% of total alcohol sales in Russia^13,17–19^.

The consumption of non-beverage alcohol is the most concerning type of alcohol consumption in Russia. This type of alcohol includes industrial surrogates such as medical alcohol, aftershaves, antifreeze, tooth powders, glues, kerosene, and brake fluid. It was easily accessible and widely consumed, primarily by males^20–22^. Alcohol surrogates were sold in pharmacies all over the country as medical tinctures, aftershaves (which were often produced with “edible” scents such as lemon, mint, or raspberry), and herbal extracts with high alcohol content^23,24^. A recent study also suggests that medicinal spirits sold in pharmacies and medicinal ethanol became the leading sources of cheap alcohol compared to other types of non-beverage alcohol after tightening regulations in 2016, which targeted perfumery and cosmetics products^25^.

The price of surrogates was usually much lower than the price of officially produced alcoholic beverages, which was an important factor in the high prevalence of consumption of non-beverage alcohol^26,27^. Researchers have also identified other important predictors of hazardous alcohol consumption in Russia. For instance, unemployment and hazardous drinking formed a vicious circle as it was identified as a boosting factor for *samogon* consumption both among males and females alike, however, the effect for males appeared to be more detrimental^26,28, 29^. Education, particularly among males, was inversely correlated with hazardous alcohol consumption^30,31^. Furthermore, materially poor individuals and those with pessimistic attitudes about their financial situation, including during the COVID-19 pandemic, were more likely to be consumers of hazardous alcohol^29,32, 33^.

Individual-level characteristics are important covariates of hazardous drinking, but they are unlikely to completely explain spatial variation in hazardous alcohol consumption. The highest levels of alcohol consumption in Russia were registered in the Far East region (primarily in Chukotka Autonomous Okrug), as well as in Siberia. The north of Russia was also significantly affected by remarkably high levels of drinking^34,35^. It is also known that in the European part of Russia, *samogon* and strong spirits such as vodka were consumed more commonly than beer and wine^36,37^. Homemade wine consumption was more common in the Southern parts of Russia due to climatic conditions suitable for grapes and other fruit production^36^.

Alcohol was more commonly consumed in rural rather than in urban areas; villages and small settlements had the highest rates of alcohol consumption, while people in cities and especially regional centers drank relatively less^38–40^. *Samogon* was normalized as an accepted part of rural culture and was widely used to facilitate social relationships^21,41^. It is estimated that rural residents consumed, on average, 17.3 L of alcohol per capita per annum, of which 14.4 L of *samogon*^31^. Contrary to samogon, non-beverage alcohol was mostly consumed in the urban areas and larger settlements with more developed industrial production and transportation infrastructure rather than in smaller settlements in the country’s rural areas^42^.

The lack of state regulations, low prices, and wide accessibility are helpful in understanding why consumption levels of hazardous alcohol were high throughout the country^43,44^, but they cannot explain the variance in hazardous drinking across Russian towns. Some studies on hazardous drinking link such alcohol consumption practices with community-level variables. For instance, the high amount of alcohol advertisements and the availability of alcohol outlets created an environment encouraging hazardous drinking in the former Soviet Union countries, including Russia^45,46^. A recent study of non-beverage alcohol consumption also demonstrated significant differences in their availability between Russian cities in 2015–2020, which may reflect varying degrees of implementation and enforcement of enacted control regulations and policies targeting non-beverage alcohol^25^.

One of the areas that attracted insufficient attention in the research of hazardous drinking in Russia is the legacy of Soviet industrialization, which entailed building more than one thousand new towns, many of which were raised as Soviet-style company towns, mono-towns, in which so-called “city-forming enterprises” accounted for a large share of total employment and also provided various social services such as housing and healthcare. After the collapse of the Soviet Union, the closure of these plants threatened entire towns with economic and social collapse and associated crisis in alcohol consumption^47,48^.

Transitional policies implemented by authorities in the 1990s might have also played a role. The speed of economic reforms, mainly through the privatization policy of major enterprises, which varied substantially across the country and among its mono-towns, could contribute to hazardous drinking. Some city-forming enterprises were fully privatized within one or two years, while for other enterprises, more gradual privatization strategies were adopted, incrementally reducing state capital over several years^49,50^.

Using data from the multilevel demographic cohort study—PrivMort, in this study, we investigate how individual and contextual factors described above—multi- vs. mono-industrial towns (defined as having a single industrial enterprise providing employment for at least 7.5% of the total population) and slow vs. fast privatization (where 90 or more percent of state shares were privatized within two consecutive years)—were associated with subsequent hazardous drinking in 30 industrial towns in the European part of Russia. This study's specific aim was to apply the mixed-effects logistic regression approach to evaluate the individual- and contextual-level covariates of hazardous drinking. We define hazardous drinking as the consumption of homemade and unofficially made alcohol and non-beverage ethanol-containing liquids not intended for human consumption.

## Methods

### Dataset

**Figure 1:**
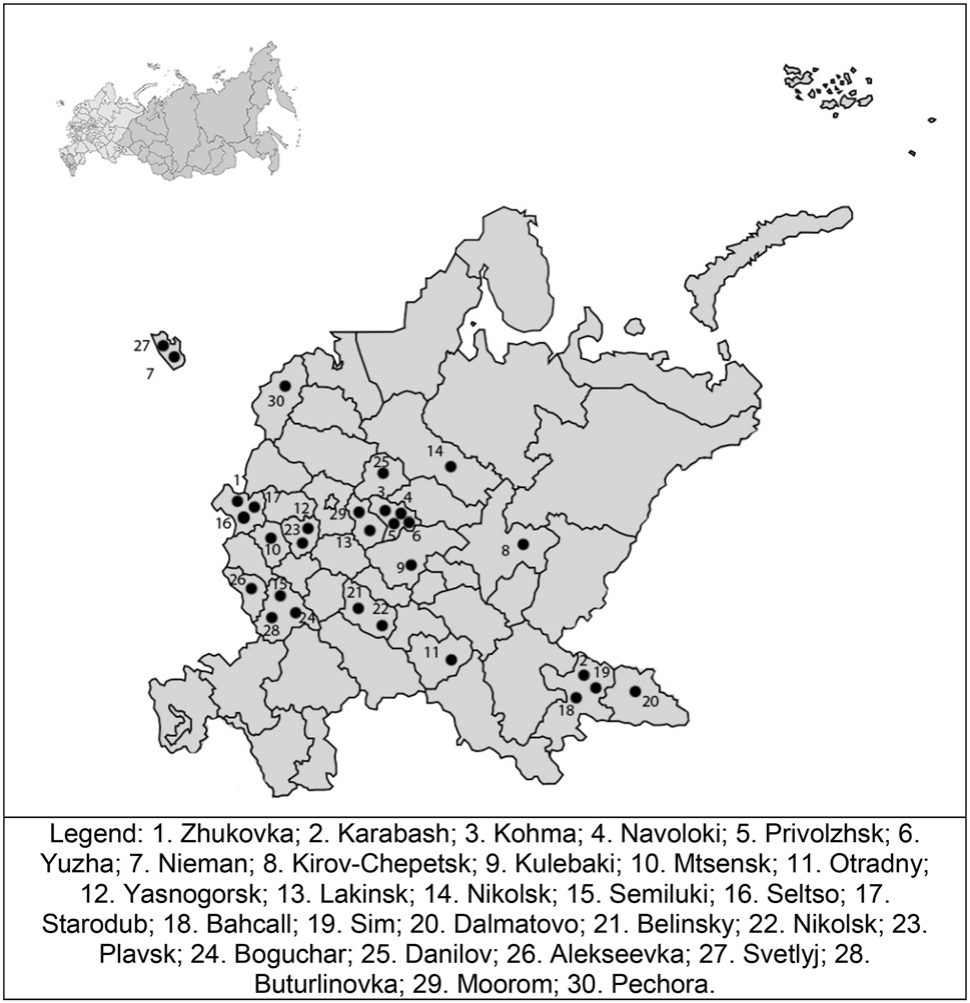
30 privatized industrial towns in the European part of Russia included in the PrivMort survey.

The analysis is based on the PrivMort data—a multi-disciplinary project investigating the post-communist morbidity and mortality crisis using a multilevel retrospective convenience cohort study^49,51, 52^. This method, originally designed for estimating demographic indicators in countries without vital registration systems, is based on an approach of data collection from informants about their relatives^53^. Between mid-September 2014 and March 2015, the PrivMort project conducted surveys in 30 towns in the European part of Russia, excluding the regions of the North Caucasus. The latter regions have dietary and alcohol consumption habits that are very different from those of the rest of Russia due to their cultural and religious traditions. The PrivMort project initially collected basic economic, demographic, and enterprise-level data on all settlements with 5000–100,000 inhabitants. A set of 30 towns, shown in Fig. 1, was selected from the pool of 539, using propensity score matching^49^. Propensity scores were calculated based on the towns’ pre-transition demographic and socio-economic conditions. To identify the health consequences of economic transformations, mono-industrial towns with fast privatization were matched to mono-industrial towns without fast privatization. Additionally, a smaller group of multi-industrial towns (where employment is distributed proportionally among several industrial enterprises) was selected, closely matching an additional set of mono-industrial towns.

Ethics approval for this study was obtained from the Ethics Committee of the University of Cambridge Department of Sociology and the European Research Council (ERC) ethics advisers. Each participant provided informed consent, and data were anonymized to prevent any potential identification of informants. Research was conducted in accordance with the ethical principles for medical research involving human subjects as described by the Declaration of Helsinki.

In the selected towns, the houses/apartments were randomly selected using a random walk method, and interviewers conducted face-to-face interviews in the selected households. Only one informant was selected from each household, even in cases when more than one family shared the same house. The screening criteria for the survey ensured that each potential informant had to be born before 1972 and had at least one family member (parents, siblings, or partners) living in the same town for a prolonged period of time during and after the transition. This criterion ensured that an informant had reached working age by 1991 and that the potential migration effect was considered. The criterion for female informants was to have at least one family member living in the same settlement in this time period, the survey excluded male informants who only had their spouses residing in the same settlement. In cases where more than one person in the household matched the screening criteria, the person whose birthday was closer to the survey date was selected for the interview. The PrivMort acquired data on informants’ parents’ (biological or step-parents), siblings’, and partners’ hazardous drinking and their other socio-demographic characteristics. Information was collected for a maximum of two siblings, the first sibling being the oldest and the second sibling being the second oldest. The collected data on partners refer to the first partners (married or long-term cohabiters) of female informants since if current partners were included, information on males who died early would be differentially excluded and, therefore, would bias results.

This collected information on the relatives was used to analyze hazardous drinking patterns and their explanations. Since the data were not collected directly, this approach, originally developed by William Brass and colleagues, is often referred to as “indirect estimation” or “Brass technique^54^.” Asking family members about their relative's hazardous drinking can provide information that could not realistically be obtained in any other way. The chance of inclusion is not uniform when the convenience cohort based on the Brass method is employed. Yet, since the survey gathered information from a diverse range of people, very few demographic groups were excluded (e.g., an older only child unmarried woman whose parents were dead). Besides, there is no evidence that this would lead to biases invalidating our findings related to hazardous drinking. The list-wise deletion of observations with missing information on hazardous alcohol consumption and other variables of interest significantly reduced our sample, and data on 20,156 relatives (55% males and 45% females) acquired from 16,680 informants (27% males and 73% females) were available for our analyses. Further details about the PrivMort survey are available in the published protocol of the study^49^.

### Questions on hazardous alcohol consumption

For homemade alcohol consumption, the survey informants were asked: “People sometimes drink homemade alcohol, that is, alcohol made by themselves or acquaintances (e.g., wine, samogon, diluted medical alcohol). Does/did your father/mother/sibling/partner ever drink such beverages? If yes, how often does/did he/she do this?” For unofficially made alcohol, the following question was asked: “People sometimes drink unofficially made alcohol, that is, alcohol made in large quantities, but not registered. Does/did your father/mother/sibling/partner ever drink such beverages? If yes, how often does/did he/she do this?” Lastly, for surrogates, informants were asked: “People sometimes drink surrogates (such as mouthwash or aftershave). Does/did your father/mother/sibling/partner ever drink such beverages? If yes, how often does/did he/she do this?” The answer options in the described questions include “often, several times a week”, “sometimes, several times a month”, “occasionally, less than once a month”, “used to drink that type of alcohol but quit” and “never”.

### Statistical analysis

To understand the covariates of hazardous alcohol consumption, we started by fitting mixed-effects logistic regressions. These models with random intercepts for both informants’ relatives and towns accounted for the dependence between relatives in the same families and towns. We assumed that random intercepts were normally distributed. Models were estimated using the Stata 17 function “melogit”. One of the main goals of this study was to find out the source of the observed cross-town variation in the consumption of hazardous alcohol. In order to test how selected types of towns, in terms of their industrial structure and privatization speed, had different levels of hazardous drinking, we fitted again mixed-effects logistic regressions in which levels 1 and 2 were, respectively, relatives and informants (families). Before we moved to town-level analysis, we first tested if the variation in hazardous drinking was accounted for by individual-level variables described below.

### Individual-level variables on relatives

Following the conventional approach of research in alcohol consumption, we divided our analytical sample by gender. Among the types of relatives for whom information was collected, fathers within male relatives and mothers within female relatives account for the substantive share of individuals with, respectively, 39% and 61%. The PrivMort survey did not collect information on female partners from male informants. A significantly higher share of female relatives (57%) than male relatives (46%) were alive at the time of the survey. The age of the individuals was collapsed into five categories: 40–49, 50–59, 60–69, 70–79, and 79 + . The variable on education was based on elementary, secondary, vocational secondary, vocational higher, and complete academic higher education. As for marital status, female relatives were much more likely to be widowed than male relatives. The survey informants were asked to report the frequency of communication between them and their relatives, including personal or via phone, internet, telegraph, or letters.

Descriptive statistics in Table 1 reveal that 35% of male relatives and 20% of female relatives were active in the labor market. A significant proportion of individuals retired at a normal age—34% of male relatives and 65% of female relatives. The PrivMort survey collected information on unemployment spells lasting for six months or longer in the 1980s, 1990s, and 2000s. This type of unemployment was experienced by about 1%, 5%, and 3% of those male relatives who were alive in these periods. For female relatives, the rates of long-term unemployment were lower at 0.6%, 2%, and 3% for the corresponding three decades. For the 1980s-2000s, we also had information on material deprivation experienced by individuals included in the sample. The share of individuals’ relatives who experienced material deprivation increased from 4% of male relatives and 5% of female relatives in the 1980s to 7% in the 1990s and decreased to 3% in the 2000s.

**Table 1:** Descriptive statistics of independent variables in 30 privatized industrial towns of Russia among informants’ relatives, %.

Variables	Male relatives	Female relatives	Variables	Male relatives	Female relatives
Type of relative			Labor market		
Father	39.1	–	Working	34.9	20.3
Mother	–	60.9	Redundant/fired	1.7	1.6
Sibling 1	14.2	27.7	Ill health	8.1	3.6
Sibling 2	6.5	11.4	Early retirement	17.0	6.4
Partner	40.2	–	Retired	34.3	64.8
Vitality status			Other reasons	4.1	3.3
Alive	45.8	56.8	Long-term unemployment		
Died in the 1980s	9.5	6.3	In the 1980s		
Died in the 1990s	16.9	12.9	Wasn’t unemployed	93.1	93.1
Died in the 2000s	19.4	16.5	Unemployed	1.1	0.6
Died in the 2010s	8.3	7.5	Wasn’ working	5.8	6.3
Age groups			In the 1990s		
40–49	10.8	5.5	Wasn’t unemployed	69.1	63.3
50–59	24.0	14.3	Unemployed	4.0	2.3
60–69	31.3	27.7	Wasn’t working	14.9	25
70–79	23.6	29.5	Wasn’t alive	12.2	9.4
> 79	10.3	23.1	In the 2000s		
Education			Wasn’t unemployed	43.5	37.1
Elementary	29.4	37	Unemployed	2.3	1
Secondary	17.5	16.2	Wasn’t working	23.7	38.3
Vocational secondary	19.8	13.4	Wasn’t alive	30.5	23.7
Vocational higher	23.1	24	Material deprivation		
Academic higher	10.2	9.4	In the 1980s		
Marital status			Never	96.0	95.4
Single	0.9	2.8	Sometimes	4.0	4.6
Married	44.3	47.6	In the 1990s		
Separated/divorced	7.8	12.5	Never	82.2	85.2
Widow/widower	6.4	37.1	Sometimes	6.4	7.0
First partner	40.4	–	Wasn’t alive	11.4	7.9
Communication			In the 2000s		
Live in the same household	14.6	17.0	Never	67.8	75.8
Every day	24	31.6	Sometimes	2.2	2.7
Once a week	29.3	29.2	Wasn’t alive	30.0	21.5
Once a month	16.1	14.0	–	–	–
A few times a year	7.6	5.5	–	–	–
Once a year	2.4	1.5	–	–	–
Less	2.3	1.3	–	–	–
No communication	3.8	–	–	–	–

## Results

### Consumption of hazardous alcohol

Table 2 presents the distribution of responses to the three questions on hazardous drinking, which also includes “don’t know” and “refuse to answer” options. Expectedly, drinking of all types of hazardous alcohol was higher among male relatives than among female relatives. Homemade alcohol was consumed more than several times a month by 12% of male relatives and only by 2% of female relatives. Unofficially made alcohol was consumed monthly or more often by 3% of male relatives and 0.3% of female relatives. Most individuals, 80% of male relatives and 93% of female relatives, have never consumed surrogates. The answer option “don’t know” is especially high for unofficially made alcohol, which suggests that many informants were not aware if alcohol consumed by their relatives was unofficially produced. Overall, our data suggest that a considerable share of Russian males engaged in hazardous alcohol consumption in the period preceding survey data collection in the first half of the 2010s.

**Table 2:** Frequencies of drinking hazardous alcohol in 30 privatized industrial towns of Russia among informants’ relatives, %.

	Male relatives			Female relatives		
	Homemade alcohol	Unofficially made alcohol	Surrogates	Homemade alcohol	Unofficially made alcohol	Surrogates
Often, several times a week	3.2	1.1	0.8	0.3	0.1	0.3
Sometimes, several times a month	8.6	2.2	1.1	1.6	0.2	0.2
Occasionally, less than once a month	22.2	3.9	1.6	16.9	0.7	0.3
Used to drink that type of alcohol but quit	9.6	3.7	1.8	6.0	0.9	0.6
Never	41.7	61.6	80.4	63.0	84.0	92.6
Don’t know	12.9	25.5	12.1	10.5	12.2	4.3
Refused to answer	1.9	1.7	2.3	1.8	1.9	1.9

**Figure 2:**
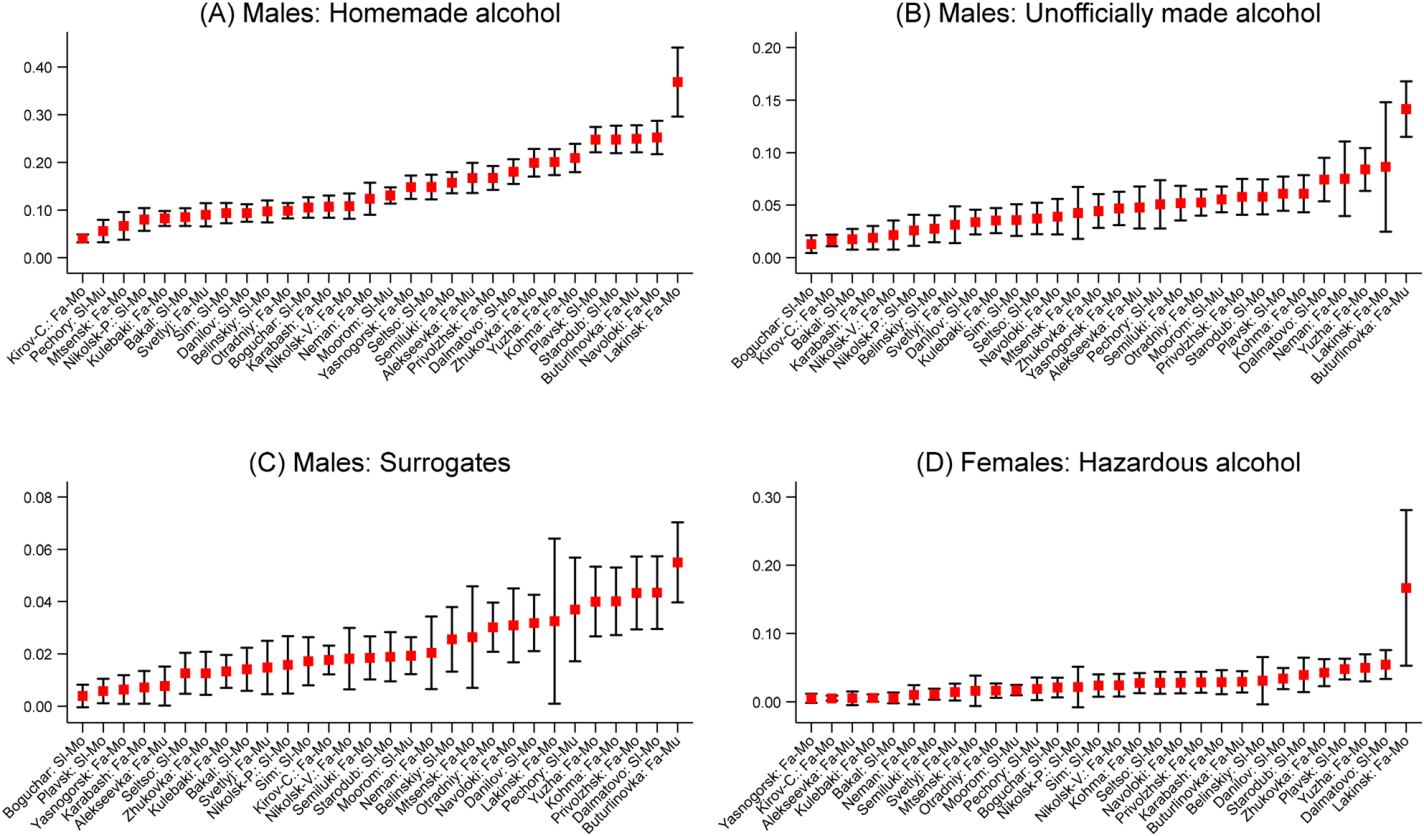
The proportion of individuals consuming hazardous alcohol in 30 privatized industrial towns of Russia among informants’ relatives. *Notes:* Bars represent 95% confidence intervals. Fa-Mo, fast mono towns; Sl-Mo, slow mono towns; Fa-Mu, fast multi towns; Sl-Mu, slow multi towns.

In order to analyze the variation in drinking in multivariable settings, for male relatives we created three binomial variables on hazardous alcohol consumption that take the value of 1 if individuals drink homemade alcohol, unofficially alcohol, and surrogates often (several times a week) or sometimes (several times a month). For females, we created only one dummy variable on hazardous alcohol consumption, which takes a value of 1 if they consumed any of the three types of hazardous alcohol. The latter procedure was necessary because the share of female relatives consuming unofficially made alcohol and surrogates was less than 0.5%, which made a meaningful analysis of its explanations unfeasible. Figure 2 presents the mean levels of consumption of homemade, unofficially made, and surrogates in 30 towns for both genders. These results suggest that there were clear differences between towns among male relatives. This is especially true for homemade and unofficially made alcohol. Kirov-Chepetsk, a town in Kirov Oblast, had one of the lowest levels of hazardous alcohol consumption. On the other end of the distribution, the highest levels of hazardous alcohol consumption were observed in Lakinsk and Buturlinovka.

### Individual-level explanations

In Table 3, mixed-effects logistic regression models for hazardous drinking with random intercepts are presented at both the settlement- and informant levels. We proceed now with the description of individual-level explanations of hazardous drinking. Starting with male relatives, we observed that partners, when compared with fathers, had odds ratios of 3.55 (p < 0.001), 5.75 (p < 0.001), and 6.33 (p < 0.001) to consume homemade alcohol unofficially made alcohol and surrogates. We also observed differences in hazardous drinking between siblings, which is in line with previous scholarship on the salience of sibship size and sibship composition in drinking behavior^55^. The vitality status mattered only for homemade alcohol consumption, with particularly high odds among those who died in the 1990s and 2000s. Among male relatives, age differences were particularly significant for the consumption of unofficially made alcohol and surrogates. Those in the 40–49 age bracket, for instance, had odds ratios of 11.8 (p < 0.001) and 6.6 (p < 0.001) to engage in the latter forms of drinking when compared with the reference age category.

**Table 3:** Odds ratios from multilevel binomial logistic regressions of hazardous drinking in 30 privatized industrial towns of Russia among informants’ relatives.

	Male relatives						Female relatives	
	Model 1: Homemade alcohol		Model 2: Unofficially made alcohol		Model 3: Surrogates		Model 4: All hazardous alcohol	
	OR	(SE)	OR	(SE)	OR	(SE)	OR	(SE)
Fixed-effects								
Type of relative (ref. fathers for male relatives, mothers for female relatives)								
1st sibling	0.68**	(0.09)	0.44**	(0.12)	0.22***	(0.10)	1.00	(0.26)
2nd sibling	0.77	(0.12)	0.84	(0.23)	0.56	(0.23)	0.85	(0.27)
Partner	3.55***	(0.66)	5.75***	(1.91)	6.33***	(2.63)	–	–
Vitality status (ref. alive)								
Died in the 1980s	1.98	(0.75)	2.26	(1.83)	3.41	(4.21)	7.42	(7.74)
Died in the 1990s	2.35**	(0.61)	1.51	(0.74)	1.48	(0.86)	1.31	(0.76)
Died in the 2000s	2.19***	(0.29)	1.26	(0.30)	1.48	(0.44)	1.34	(0.39)
Died in the 2010s	1.89***	(0.30)	1.48	(0.42)	0.79	(0.31)	1.51	(0.51)
Age groups (ref. more than 79)								
40–49	2.58***	(0.63)	11.83***	(6.11)	6.59**	(4.04)	1.74	(0.96)
50–59	2.35***	(0.48)	7.51***	(3.44)	2.62	(1.39)	2.00	(0.85)
60–69	1.63**	(0.28)	5.22***	(2.14)	2.73*	(1.21)	1.37	(0.50)
70–79	1.41*	(0.22)	2.29*	(0.84)	1.28	(0.51)	1.83	(0.58)
Marital status (ref. married)								
Single	0.45	(0.21)	0.76	(0.62)	2.49	(2.09)	0.37	(0.30)
Separated/divorced	1.69***	(0.21)	2.51***	(0.55)	1.58	(0.52)	1.85*	(0.46)
Widow/widower	1.33*	(0.18)	1.44	(0.38)	0.60	(0.25)	1.59*	(0.33)
Education (ref. academic higher)								
Elementary	2.96***	(0.55)	5.54***	(2.32)	0.95	(0.38)	3.61**	(1.52)
Secondary	2.26***	(0.43)	4.14***	(1.73)	0.77	(0.33)	3.21**	(1.34)
Vocational secondary	2.27***	(0.42)	4.11***	(1.71)	1.16	(0.46)	1.80	(0.78)
Vocational higher	1.35	(0.25)	2.95**	(1.22)	0.59	(0.25)	1.34	(0.55)
Communication								
In the same household	1.07	(0.14)	1.22	(0.30)	2.32**	(0.74)	1.36	(0.38)
Once a week	1.08	(0.12)	1.10	(0.24)	1.27	(0.39)	1.23	(0.28)
Once a month	1.16	(0.16)	1.22	(0.31)	1.70	(0.59)	1.63	(0.45)
A few times a year	1.44*	(0.24)	1.70	(0.52)	1.11	(0.51)	2.13*	(0.79)
Once a year	1.90*	(0.48)	1.85	(0.81)	1.84	(1.12)	0.43	(0.48)
Less	3.63***	(0.94)	2.71*	(1.23)	2.89	(1.68)	3.70*	(2.35)
No communication	3.17***	(0.82)	4.33***	(1.77)	2.73*	(1.34)	–	–
Labor market status (ref. working)								
Redundant/fired	1.94**	(0.47)	5.84***	(2.25)	4.00**	(2.10)	1.41	(0.83)
Ill health	0.97	(0.16)	0.96	(0.32)	1.31	(0.59)	3.65***	(1.44)
Early retirement	1.25	(0.20)	2.11**	(0.60)	2.54*	(1.04)	0.98	(0.45)
Retired	0.78*	(0.10)	1.28	(0.31)	2.13*	(0.75)	1.02	(0.28)
Not working for other reasons	1.53*	(0.32)	4.05***	(1.35)	4.12**	(1.87)	0.93	(0.69)
Long-term unemployment (ref. not unemployed)								
Unemployed in the 1980s	2.21*	(0.69)	4.13**	(1.87)	8.33***	(4.80)	3.02	(2.10)
Not working in the 1980s	0.61*	(0.15)	0.56	(0.26)	0.31	(0.23)	0.37	(0.21)
Unemployed in the 1990s	1.86**	(0.38)	1.08	(0.39)	1.68	(0.75)	1.72	(0.74)
Not working in the 1990s	0.98	(0.11)	1.05	(0.23)	0.79	(0.22)	0.62	(0.16)
Unemployed in the 2000s	1.99**	(0.52)	1.64	(0.74)	1.48	(0.85)	5.90***	(3.07)
Not working in the 2000s	1.25	(0.16)	2.22***	(0.53)	2.25**	(0.71)	0.72	(0.20)
Material deprivation (ref. never)								
Sometimes in the 1980s	1.21	(0.25)	1.43	(0.48)	0.51	(0.26)	1.36	(0.55)
Sometimes in the 1990s	1.24	(0.23)	2.43**	(0.73)	1.63	(0.65)	1.35	(0.51)
Sometimes in the 2000s	2.63***	(0.67)	2.50*	(0.97)	7.38***	(3.84)	3.05*	(1.39)
Intercept	0.01***	(0.00)	0.00***	(0.00)	0.00***	(0.00)	0.00***	(0.00)
Random-effects								
Town-level variance	0.50	(0.16)	0.61	(0.23)	0.79	(0.34)	0.45	(0.20)
Family-level variance	1.96	(0.45)	2.35	(1.14)	3.58	(1.71)	2.36	(1.33)
Model statistics								
ICC on town-level								
Null model	0.09	(0.02)	0.06	(0.02)	0.07	(0.02)	0.09	(0.03)
Full model	0.09	(0.02)	0.10	(0.03)	0.10	(0.04)	0.07	(0.03)
ICC on family-level								
Null model	0.37	(0.03)	0.40	(0.05)	0.43	(0.05)	0.43	(0.08)
Full model	0.43	(0.05)	0.47	(0.11)	0.57	(0.10)	0.46	(0.12)
BIC	7561.92		2762.42		1994.62		2080.54	
Number of towns	30		30		30		30	
Number of families	8878		7656		9136		7544	
Number of individuals	10,711		9164		11,107		9049	

*Notes:* ***, ** and * denote statistical significance at the 0.001, 0.01, and 0.05 levels, respectively.

For female relatives and surrogate-drinking male relatives, marital status only marginally explained the variance in the dependent variables. Separated/divorced male relatives had much higher odds ratios for consuming both homemade (1.7, p < 0.001) and unofficially made (2.5, p < 0.001) alcohol. In addition, separated/divorced (1.8, p < 0.05) and widowed (1.6, p < 0.05) female relatives were more likely to drink hazardous alcohol. Individuals’ educational attainment was an important explanation of alternative alcohol consumption. Male relatives with elementary, secondary, and vocational secondary educational attainment had odds ratios of 5.5 (p < 0.001), 4.1 (p < 0.001), and 4.1 (p < 0.001) to consume unofficially made alcohol. Female relatives with elementary (3.6, p < 0.01) and secondary (3.2, p < 0.01) education were also more likely to engage in alternative alcohol consumption. Similarly, we found that social contacts had an important effect on drinking patterns. Those male relatives who communicated with female informants less than once a year had odds ratios of 3.3 (p < 0.001) and 2.7 (p < 0.05) to drink homemade and unofficially made alcohol. For all three types of hazardous alcohol, the likelihood of drinking was especially high for those male relatives who did not communicate with the survey informants, as they had the odds ratios of 3.2 (p < 0.001), 4.3 (p < 0.001), and 2.7 (p < 0.05) for three types of alternative alcohol consumption.

Another important explanation of hazardous drinking was individuals’ labor market status. Compared to working male relatives, those who were made redundant or were fired from their work had significantly higher odds of hazardous drinking, especially in regard to unofficially made alcohol (OR 5.8, p < 001) and surrogates (OR 4.0, p < 01). In turn, females who did not work due to ill health had an odds ratio of 3.6 (p < 0.01) to drink hazardous alcohol. The results suggest that there were long-term consequences of life-course events as long-term unemployment among male relatives in the 1980s exhibited a statistically significant effect on all types of hazardous alcohol consumption with odds ratios of 2.2 (p < 0.05), 4.1 (p < 0.01) and 8.3 (p < 0.001) to drink homemade alcohol, unofficially made alcohol and surrogates. Male relatives who were unemployed in the 1990s (OR 1.9, p < 0.01) and the 2000s (OR 2.0, p < 0.01) were also more likely to drink homemade alcohol. For female relatives, the only significant association between long-term unemployment and hazardous drinking stemmed from the most recent decade, with an odds ratio of 5.9 (p < 0.001).

Finally, we looked on the second variable for which information was available for three consecutive decades—individuals’ material deprivation. We found an expected relationship between experiencing deprivation and consuming hazardous alcohol, but this effect was primarily manifested in the 2000s. Materially deprived male relatives in the 2000s had odds ratios of 2.6 (p < 0.001), 2.5 (p < 0.05), and 7.4 (p < 0.001) to drink homemade alcohol, unofficially made alcohol, and surrogates. In addition, male relatives who experienced deprivation in the 1990s were also more likely to consume unofficially made alcohol (2.4, p < 0.01). The effect of material deprivation in the 2000s was significant for female relatives who had an odds ratio of 3.1 (p < 0.05) to drink at least one type of hazardous alcohol.

### Intraclass correlation coefficients

We calculated two intraclass correlation coefficients (ICC) for the described mixed-effects logistic regressions. The first is the ICC at the town level that showed the correlation between consumption of alternative alcohol in the same town. The second is the ICC at the families-within-towns level, which showed the correlation between the consumption of alternative alcohol in the same family and town. The results suggest that, controlling for individual-level covariates, hazardous drinking among individuals was only slightly correlated within the same towns, but it was highly correlated within the same families and towns. Estimated ICCs indicated that family and town random effects composed 43%, 47%, and 57% of the total residual variance for consumption of, respectively, homemade alcohol, unofficially made alcohol, and surrogates. Family and settlement random effects for female relatives composed 46% of the total residual variance. One would expect that family-level correlation coefficients could be higher for homemade alcohol as it, by definition, implies that consumed alcohol is made within families, but our estimations showed that for male relatives, association within the family was higher for consumption of unofficially made alcohol and surrogates.

Lastly, we compared ICC values from null models without any individual-level variables and full models with all individual-level variables, which showed that instead of decreasing, the variance in hazardous alcohol consumption among towns stayed unchanged (e.g., for homemade alcohol consumption among male relatives) or even increased (e.g., for all other forms of hazardous drinking among male relatives). This suggested that not only the descriptive results on differences between towns depicted in Fig. 1 could not be explained by individual-level composition of these towns, but also these differences were even more pronounced when individual-level characteristics were taken into account.

### The types of towns and consumption of hazardous alcohol

In this section, we explore if the variance in hazardous drinking was also explained by the different patterns of industrialization and privatization of 30 towns where the survey was conducted. For this purpose, in Table 4, we compared hazardous drinking between slow- and fast-privatized multi-towns, and slow- and fast-privatized mono-towns by fitting mixed-effects logistic regressions. In Panel 1, which accounts for individual-level characteristics, we see statistically significant differences between the four considered types of towns. Individuals in fast-privatized multi-towns had an odds ratio of 3.3 (p < 0.001) to drink homemade alcohol when compared with slow-privatized multi-towns, while in slow- and slow-privatized mono-towns, odds ratios equaled 2.3 (p < 0.001) and 1.8 (p < 0.01), respectively. Furthermore, in fast-privatized multi-towns, individuals had odds ratios of 2.3 (p < 0.05) and 4.6 (p < 0.01) to drink unofficially made alcohol and surrogates when compared to individuals in slow-privatized multi-towns. We also observed that female relatives in slow-privatized mono-towns had an odds ratio of 2.3 (p < 0.05) to consume hazardous alcohol.

**Table 4:** Odds ratios from multilevel binomial logistic regressions of hazardous drinking in four different types of privatized industrial towns of Russia among informants’ relatives, %

	Panel 1: All relatives of interviewed individuals			
	Male relatives			Female relatives
	Model 1: Homemade alcohol	Model 2: Unofficially made alcohol	Model 3: Surrogates	Model 4: Alternative alcohol
Slow privatized multi-towns	1.00	1.00	1.00	1.00
Fast privatized multi-towns	3.28 (0.76)***	2.27 (0.77)*	4.57 (2.47)**	1.14 (0.51)
Slow privatized mono-towns	2.31 (0.47)***	0.69 (0.20)	1.81 (0.89)	2.35 (0.84)*
Fast privatized mono-towns	1.76 (0.35)**	0.85 (0.24)	1.84 (0.88)	1.14 (0.40)
Number of individuals	10,711	9164	11,107	9049

	Panel 2: Only those relatives living in the analyzed towns in the 1980s-1990s			
	Male relatives			Female relatives
	Model 5: Homemade alcohol	Model 6: Unofficially made alcohol	Model 7: Surrogates	Model 8: Alternative alcohol
Slow privatized multi-towns	1.00	1.00	1.00	1.00
Fast privatized multi-towns	3.61 (1.01)***	2.79 (1.07)**	7.85 (5.09)**	2.75 (1.40)*
Slow privatized mono-towns	2.27 (0.53)***	0.66 (0.21)	1.98 (1.11)	3.20 (1.32)**
Fast privatized mono-towns	1.54 (0.34)	0.78 (0.23)	2.18 (1.19)	1.51 (0.63)
Number of individuals	7840	6574	8067	6464

*Notes:* ***, ** and * denote statistical significance at the 0.001, 0.01, and 0.05 levels, respectively. Standard errors are shown in parentheses. Models account for all variables shown in Table 3.

To more accurately account for the possible effects on hazardous alcohol consumption related to the type of industry and the speed of privatization, it is important to include in regression models only those relatives who lived in the towns in question in the 1980s and the 1990s. For this purpose, we eliminated all relatives who immigrated to those areas after the post-communist transition had started or those who left the towns in question in the 1990s. The results from using this new and more conservative sample are shown in Models 5–8 in Table 4. They are substantially similar to the findings in Models 1–4 when all individuals were included in the analyses. Male relatives in fast-privatized multi-towns, slow-privatized mono-towns, and fast-privatized mono-towns in Model 5 had odds ratios of 3.6 (p < 0.001) and 2.3 (p < 0.001) to drink homemade alcohol. Male relatives in fast-privatized multi-towns were also much more likely to drink unofficially made alcohol (OR 2.8, p < 0.01) and surrogates (OR 7.8, p < 0.01). In Model 8, female relatives in fast-privatized multi-towns and slow-privatized mono-towns, respectively, had odds ratios of 2.7 (p < 0.05) and 3.2 (p < 0.01) to drink hazardous alcohol.

**Figure 3:**
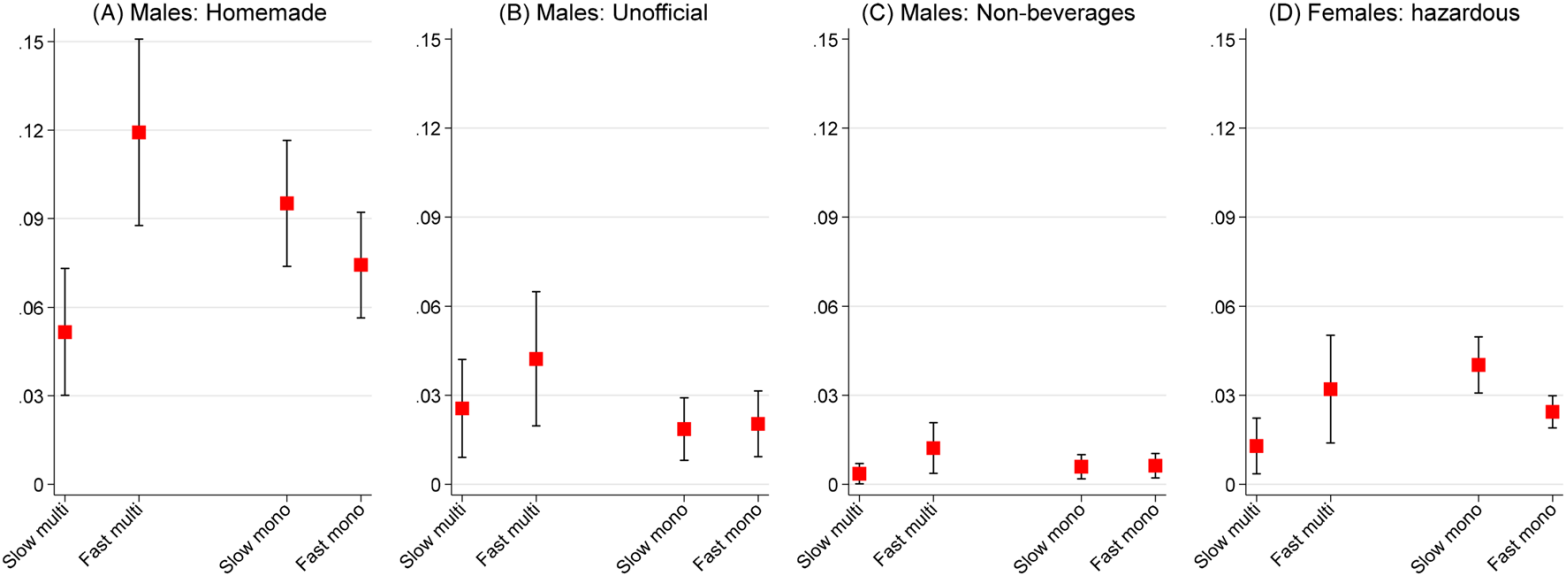
Predictive margins from multilevel binomial logistic regressions of hazardous drinking in four different types of privatized industrial towns of Russia among informants’ relatives, %. *Notes:* Bars represent 95% confidence intervals.

Lastly, to determine how the specific levels of hazardous drinking varied among the four types of towns considered, we also calculated predictive margins after fitting mixed-effects logistic regressions for three types of hazardous alcohol consumption. The derived results in Fig. 3 indicate that controlling for individual-level explanations, the highest levels of hazardous alcohol consumption, particularly of homemade and unofficially made alcohol, were observed in fast-privatized multi-towns, while the lowest incidents of hazardous drinking were observed in slow-privatized multi-towns. For male relatives, these differences were statistically significant only for homemade alcohol consumption, while for female relatives’ consumption of hazardous alcohol was somewhat higher in slow- and fast-privatized mono-towns than in slow-privatized multi-towns. The results suggested that hazardous alcohol consumption was indeed associated with industrial structure and speed of privatization of towns where the PrivMort survey was conducted.

## Discussion

In this study, we investigated the individual- and contextual-level explanations of hazardous alcohol consumption in 30 slow- and fast-privatized industrial towns in Russia. One of the main strengths of this study was that, unlike the conventional surveys on hazardous alcohol consumption, we were able to account for individuals who were deceased, among other reasons, due to hazardous alcohol consumption. Our estimates for the sample of informants’ relatives suggest that about 12%, 3%, and 2% of male relatives consumed, respectively, homemade alcohol, unofficially made alcohol, and surrogates at least several times a month up until the period when the survey was conducted. Among female relatives, the consumption of homemade alcohol a few times a month was around 2%, while only a small fraction of female relatives drank unofficially made alcohol and surrogates. Descriptive statistics also suggested that the differences in hazardous alcohol consumption between towns were significant among male relatives, especially for homemade and unofficially made alcohol.

We first assumed that one of the reasons why we observed the differences in hazardous alcohol consumption between survey towns could be that they diverged from each other by their socio-demographic and socio-economic composition. Among the analyzed towns, Kirov-Chepetsk had one of the lowest levels of hazardous drinking. It is difficult to speculate what the causes of such a low level of drinking were, but the town itself was considered to be a well-developed urban center with a vibrant cultural and educational life. The highest levels of hazardous alcohol consumption, in turn, were observed in Lakinsk and Buturlinovka. The former is a small town located in Vladimir Oblast. It was populated by about 19 thousand individuals in 1989, with a declining population thereafter. The town also had a large Brewery^56^. Lakinsk was an outlier, especially regarding hazardous alcohol consumption among females. Further, Buturlinovka is located in Voronezh Oblast and was described as having an unfavorable socio-economic situation and low indicators of the level of commercial services^57^. It is also the home of the Buturlinovka air base, which has been actively used by the Russian armed forces after the invasion of Ukraine in 2022. Both towns are situated in the regions of Russia with high availability of non-beverage alcohol and illicit alcoholic beverages, as was identified in the availability survey in the cities of Voronezh and Rossosh (Voronezh Oblast) and Petushki (Vladimir Oblast) in 2015–2020^25^.

It is important to highlight that the consumption of hazardous alcohol may significantly differ in various towns depending on their proximity to federal highways, roads, and railways, which are extensively used by shadow alcohol businesses for the trafficking of unrecorded ethanol used for the production of unofficially produced, non-beverage, and surrogate alcohol. Even for the production of *samogon*, the cheap industrially produced ethanol, diverted from the legal market, was often used to increase *samogon’s* alcoholic strength or to produce fake *samogon*, which requires an appropriate supply of industrial ethanol via convenient transportation routes. In this regard, the situation observed in Buturlinovka and Lakinsk could be partially explained by their close proximity to federal highways M-7 “Volga” (Lakinsk) and M-4 “Don” (Buturlinovka), and both towns are well connected to the rest of the country by railway. The latter is supported by a study conducted in the Irkutsk region that showed that the incidence of poisonings with ethanol and alcohol surrogates was higher in towns situated along the federal highway P255 “Siberia”^42^.

Our analyses of individual-level variables indicate that the consumption of hazardous alcohol was highest among male relatives aged 40–49, male relatives and female relatives with elementary and secondary education, and males and females who were separated or divorced. The observed age differences in hazardous drinking might be related to the generational shift in Soviet Russia when alcohol consumption moved from the public to the private sphere and became an acceptable norm^58^. If before, people consumed alcohol as a collective social act, the intake of alcohol in isolation of private homes started to increase in the pre-television era and likely further contributed to hazardous drinking in Russia^59^. It might also be the case that homemade alcohol was perceived as less dangerous by less educated individuals than official cheap alcohol as it was “homemade” and hence perceived to be more “natural.”

We also revealed that hazardous drinking was higher among those male relatives who lost their jobs, retired early, and did not work, those females who did not work due to ill health, and those male relatives and female relatives who had experienced spells of long-term unemployment and material deprivation in the 1980s to 2000s. These findings corroborate the previous scholarship. Material deprivation increased hazardous drinking as non-beverage alcohol was significantly cheaper^60,61^. Financial strain, especially among the elderly, might result in them producing and selling *samogon* as a means of diversifying their income, which increases the accessibility of *samogon* and reduces its market price due to the increased supply^62,63^. In addition, we found that hazardous alcohol consumption was higher among those male and female relatives who rarely communicated with their relatives—our survey informants. The latter could be both the cause and the effect of hazardous drinking, as people who consume hazardous alcohol might be shunned by or seek voluntary isolation from their relatives.

Nonetheless, despite revealing significant effects of individual-level variables on hazardous drinking, our estimations suggested that the composition of towns did not account for the reduction of variance in hazardous alcohol consumption between these territorial units. Controlling for individual-level explanations, we tested how macro-level characteristics were associated with alcohol consumption in towns where our data were collected. Using mixed-effects logistic regressions, we found that when compared with slow-privatized multi-industrial towns, the consumption of homemade alcohol was significantly higher among male relatives in fast-privatized multi-industrial towns as well as in slow-privatized mono-industrial towns, while consumption of unofficially made alcohol and surrogates among male relatives was also higher in fast-privatized multi-industrial towns. For female relatives, we showed that consumption of hazardous alcohol was highest in fast-privatized multi-towns and slow-privatized mono-towns. These findings overall corroborated previous evidence linking fast privatization and liberalization policies with the worsening well-being and other social outcomes during the post-communist transition^48,64–66^.

The results of our analyses can have some policy implications. Evidence suggests that a series of alcohol policies that the government adopted after 2000 contributed to the downward trend in hazardous alcohol consumption in the country. Among other policies, these measures included eliminating the various markets of unrecorded alcohol, reducing alcohol misuse and alcohol use disorders, and putting emphasis on shifting drinking patterns in the general population of the country. Through various measures introduced in 2005–06 alcohol policies mainly targeted the reduction of the proportion of unrecorded alcohol. However, policy measures since 2011 have been directed at consumers and their individual behaviors by increasing alcohol excise taxes, raising the minimum unit price, and reducing the availability of retailed alcohol. Furthermore, hazardous drinking was included as a risk factor in a series of legislative acts that aim to promote a healthy lifestyle in the population at national and regional levels^67^. One of the most important problems in this regard remains the uneven implementation and differential enforcement of the above-described alcohol-related policies in various territorial units of Russia, which may significantly affect the varying availability and consumption of hazardous alcohol in the 30 towns included in our study. For instance, the differences in implementing unrecorded alcohol control policies between various regions and towns were vividly illustrated in the 2015–2020 availability survey^25^.

Despite the described policy initiatives in the direction of tighter control of alcohol, hazardous drinking in Russia continues to be largely perceived as a problem of individuals who might require the help of narcologists^45^. Since the situation with hazardous drinking exceeds individuals and is a mass problem, there is a need for further policy measures to address this issue^27,68^. Affecting the problems of unemployment and material deprivation by means of economic progress could potentially reduce hazardous alcohol consumption. Educational campaigns on the potential harmful consequences of hazardous alcohol consumption and more restrictive regulations of the alcohol market in Russia might also reduce hazardous drinking. Arguments have been advanced in previous studies that additional policies restricting the availability and low prices of unofficial alcohol need to be made a priority in Russia and in the broader Eastern European region^69^. On the town level, since our study showed that hazardous drinking was higher in multi-industrial towns that experienced fast privatization as well as in some mono-industrial towns, more careful consideration of the social consequences of economic policies could be important for the prevention of socio-economic problems and associated hazardous alcohol consumption. Investing more in local infrastructure and redistributive policies could also have a significant impact on hazardous drinking^70^.

Our study is based on a complex approach to data collection and has at least four important limitations. First, the data were collected in the European part of Russia, which, despite hosting 80 percent of the country’s total population, still is culturally and economically different from the rest of the country. Second, questionnaires aimed at finding out about past events and behaviors can result in recall bias. The survey administrators have tried to account for this by introducing auxiliary discussions with the informants about major events and occurrences, which could help them remember the past more clearly. Third, as non-respondents in our random selection of the houses/apartments were likely to have lower socioeconomic status and worse health, and since their relatives were likely to be of similar background and have similar health status, the convenience cohort is likely to be wealthier and healthier compared to the general population. This also implies that our sample might have omitted the heaviest drinkers, whose relatives were also more likely to consume hazardous alcohol. This, however, should not affect the investigated associations within our study. In addition, using informants' relatives instead of informants reduces the potential effect of non-response bias.

Fourth, our study could not assess the degree of enforcement and the quality of implementation of the previous and newly enacted state control policies targeting hazardous alcohol consumption in 30 surveyed towns of the European part of Russia, and this may be an important unmeasured confounding factor affecting the results of the study. The different degrees of law enforcement activity and corruption of civil authorities may have significantly undermined the implementation of policies regulating alcohol consumption in the analyzed towns, leading to observed differences in hazardous drinking between them^71^.

Finally, more research is needed to understand how hazardous drinking develops through time in connection to various individual and town-level characteristics. The Russian invasion of Ukraine in February 2022 could be an important event to explore in terms of its implications for hazardous alcohol consumption in the years to come, especially in industrial towns located close to the Ukrainian border.

## Data Availability

The data used in this study can be obtained through the Open Science Framework.

## Code availability

All analyses were conducted in Stata 17 (StataCorp, College Station, 369 TX). The code used to generate the results can be obtained through the Open Science Framework.
